# Hippocampal Neuronal Activity Preceding Stimulus Predicts Later Memory Success

**DOI:** 10.1523/ENEURO.0252-22.2023

**Published:** 2023-02-13

**Authors:** Soyeon Jun, June Sic Kim, Chun Kee Chung

**Affiliations:** 1 Neuroscience Research Institute, Seoul National University College of Medicine, Seoul, South Korea, 03080; 2 Department of Brain and Cognitive Sciences, Seoul National University, Seoul, South Korea, 08826; 3 Research Institute of Basic Sciences, Seoul National University, Seoul, South Korea, 03080; 4 Department of Neurosurgery, Seoul National University Hospital, Seoul, South Korea, 03080

**Keywords:** hippocampus, memory, neuronal activity, prediction, single-unit activity

## Abstract

Hippocampal neuronal activity at a time preceding stimulus onset affects episodic memory performance. We hypothesized that neuronal activity preceding an event supports successful memory formation; therefore, we explored whether a characterized encoding-associated brain activity, viz. the neuronal activity preceding a stimulus, predicts subsequent memory formation. To address this issue, we assessed the activity of single neurons recorded from the hippocampus in humans, while participants performed word memory tasks. Human hippocampal single-unit activity elicited by a fixation cue preceding words increased the firing rates (FRs) and predicted whether the words are recalled in a subsequent memory test; this indicated that successful memory formation in humans can be predicted by a preceding stimulus activity during encoding. However, the predictive effect of preceding stimulus activity did not occur during retrieval. These findings suggest that the preparative arrangement of brain activity before stimulus encoding improves subsequent memory performance.

## Significance Statement

Hippocampal neuronal activity at a time preceding stimulus onset affects episodic memory performance. This predictive effect may reflect encoding attentional selection, providing an excitatory advantage to the hippocampal cells involved in coding the cue.

## Introduction

Human brain activity elicited by initially faced stimulus events is a critical determinant of whether an item will be successfully memorized later (Sanquist et al., 1980; Rugg and Coles, 1995; Paller and Wagner, 2002). In previous studies, researchers have focused on memory-predictive brain activation during stimulus presentation using the “subsequent memory effect” (SME) approach. It is classically known as the neural activity difference between items subsequently remembered and items are forgotten while encoding during stimulus presentation (Paller et al., 1987; Paller and Wagner, 2002). In this approach, the recorded neural activity during the encoding period is classified based on whether these items are remembered or forgotten in the subsequent memory test. Brain activities differentiating between subsequently remembered and forgotten items encoding-related activity and fleeting neural activity induced by individual items are used as a neural marker in successful memory formation. Although findings from this approach have yielded important insights into the neural mechanisms underlying successful memory encoding (Jun et al., 2019, 2020, 2021), studies are limited concerning the exploration of encoding-correlated brain activity lined up by stimulus events.

Learning from novel events is dependent on various brain processes and mechanisms. Thus, in reality, experiences and memorandum do not exist separately; rather, they proceed on a sequential continuum. Specifically, present events are, in some measure, a consequence of past events and could provide cues regarding future events. Thus, at any given moment in time, the neural activity resulting from experiences will influence the memory of the events faced. The use of functional magnetic resonance imaging (fMRI) suggests that brain activity occurring immediately before encoding can predict subsequent memory performance in semantic preparatory task sets, improving the encoding of words processed deeply (Otten et al., 2006; Guderian et al., 2009).

Additionally, neural activity is tonically maintained across a succession of stimulus events involved in successful episodic memory encoding (Otten et al., 2002). In addition, Park and Rugg (2010) investigated human neuronal activity in the hippocampus and found that attention to encoding predicts subsequent memory in the medial temporal lobe in the prefrontal and anterior cingulate cortices. These results show that the hippocampal predictive preonset spiking effect is much more robust (Urgolites et al., 2020). Further investigation is needed to create additional knowledge concerning the diverse neural mechanisms supporting hippocampus-dependent memory encoding. However, only a few studies have investigated the effect of predictive neuronal activity’s effect on subsequent memory performance and successful memory formation, specifically in hippocampal-dependent activity.

The present study explored whether a characterized encoding-associated brain activity predicts subsequent memory formation. We used the “preceding stimulus subsequent memory effect” (pre-SME) procedure in human hippocampal single neuronal activity during encoding and retrieval stimulus interval. Additionally, we investigated whether there is a characterization of hippocampus-dependent task effects in word item memory or word associative memory (Staresina et al., 2019; Jun et al., 2020) on hippocampal neuronal activities preceding a stimulus.

## Materials and Methods

### Subjects and microelectrode recording

**Figure 1.**
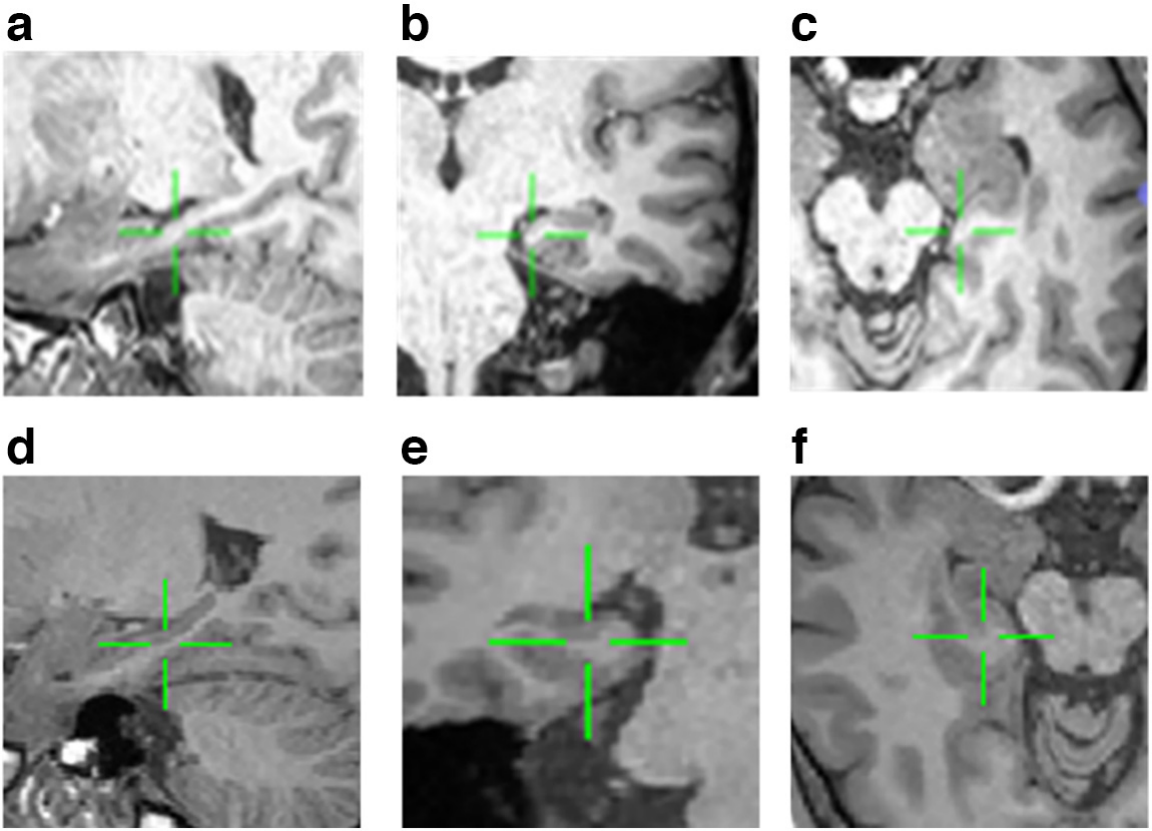
Electrode localization with structural magnetic resonance images. Examples of hippocampal microelectrodes from two patients are shown (see Table 1 for demographic information). The patient’s left brain is shown on the left side in the axis plane. All images are T1 images recorded using Sigma 3-tesla scanner (GE). Green arrows indicate electrodes inserted in the hippocampal subfield of Cornu Ammonis (CA3 (above) and CA1 (bottom)). ***a***, Sagittal image of the left hippocampal CA3. ***b***, Coronal hippocampal CA3. ***c***, Axial hippocampal CA3. ***d***, Sagittal image of the left hippocampal CA1. ***e***, Coronal CA1 image. ***f***, Axial CA1 image.

Four male patients with epilepsy aged 25–64 year (M = 39.7, SE = 17.3) were implanted with hybrid depth electrodes for seizure monitoring. We assessed all individuals using a typical neuropsychological test (Table 1). Nine microwires (eight high-impedance recording electrodes and one low-impedance reference; Ad-Tech) projecting from the shaft of the depth electrodes were used to record signals from medial temporal lobe neurons. Electrode locations (Fig. 1) were selected following the clinical criteria. Target electrode locations were confirmed using a human hippocampal atlas created using a postoperative computed tomography (CT) image that was reconstructed from individual preoperative magnetic resonance (MR) images using CURRY 8.0 (Compumedics Neuroscan). MR images were collected using a Sigma 1.5-tesla scanner (GE). A neuroradiologist identified each electrode contact using a thin postimplant CT scan section. The brain model and implanted electrodes were reconstructed from individual preoperative MR and postoperative CT images using CURRY 7.0 (Compumedics Neuroscan). A neuroradiologist and neurosurgeon then confirmed the contact of the hippocampal electrodes. We further investigated an automatic segmentation for the hippocampal subfield using an open-source software package (Fischl, 2012) with T1-weighted images (Iglesias et al., 2015) and annotations using Freeview. Electrodes that could be contained in the hippocampus or amygdala were included in this experiment. All individuals had adequate recognition and distinguishable neuronal spiking activities.

**Table 1:** List of patient demographics, pathology, and neuropsychological evaluation

Subject	Age	Sex	Epilepsy diagnosis	WAIS-IV					WMS-R
				VCI	PRI	WMI	PSI	FSIQ	MQ
Sub 1	30–40	M	RTLE	116	86	98	84	95	66
Sub 2	20–30	M	LTLE	100	107	98	55	90	73
Sub 3	60–70	M	RTLE	114	94	112	92	103	77
Sub 4	50–60	M	LTLE	72	84	78	72	70	56
Average	39.7 (17.3)	-	-	101 (17.6)	93 (9.0)	97 (12.1)	76 (13.9)	90 (12.2)	68 (8.0)

Intelligence was measured with the Korean Wechsler Memory Scale (K-WMS) and memory with the Wechsler Memory Scale (WMS). RTLE, right temporal lobe epilepsy; LTLE, left temporal lobe epilepsy; VCI, verbal comprehension index; PRI, perceptual reasoning index; WMI, working memory index; PSI, processing speed index; FSIQ, full scale IQ; MQ, memory quotient.

### Experimental design and single-unit identification

**Figure 2.**
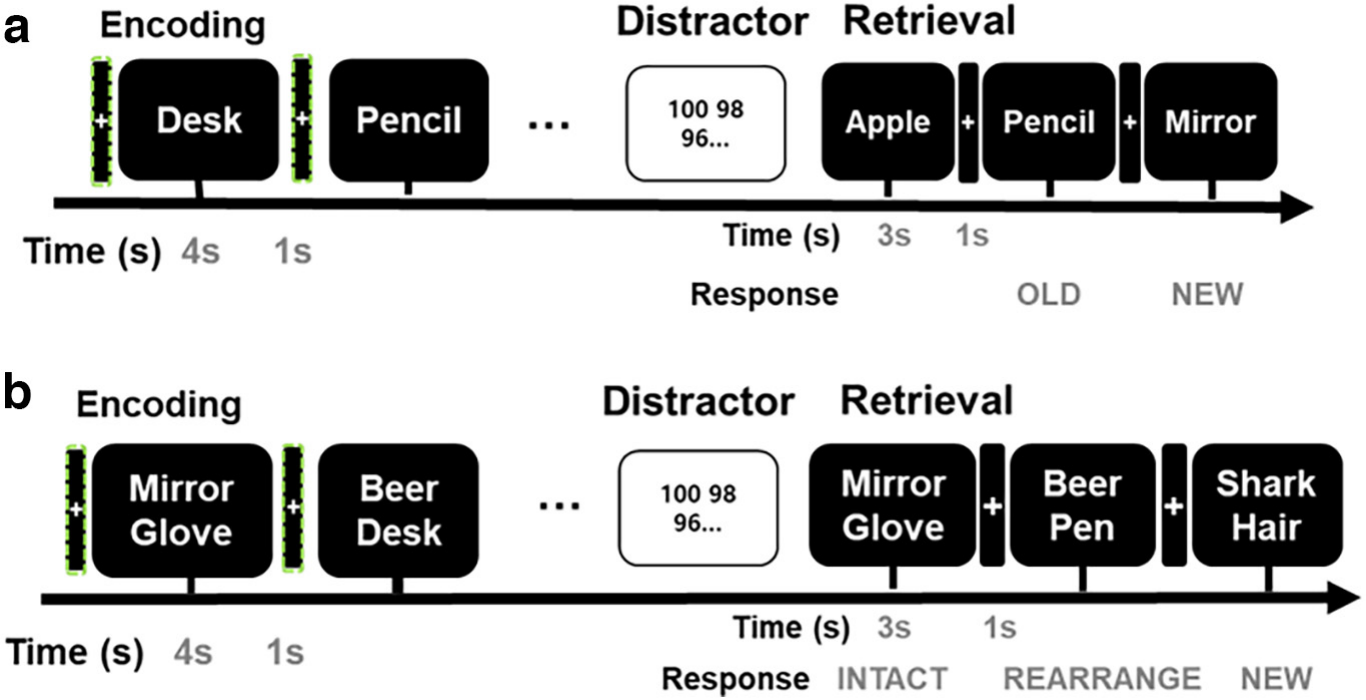
Word memory tasks. ***a***, Example of the timeline of the visual item memory task and (***b***) associative memory task. Memory tasks comprised three successive stages encoding, distractor, and retrieval. Each patient sequentially completed words items during encoding, which was followed by a white fixation cue with a black screen for 1 s. The green dashed line box indicates the preceding stimulus interval.

We recorded single neuronal activities in four individuals and had individuals encode visually presented word memory tasks using STIM2 software (Compumedics Neuroscan). The patients had the same memory task (Fig. 2) as our previous study using the word memory tasks (Jun et al., 2019, 2020, 2021). Behavioral data were analyzed with SPSS 23 (IBM). Extracellular neural activity was recorded using 40-μm micro-wires implanted in a depth electrode and inserted in the hippocampus and amygdala. Spiking and intracranial electroencephalography (iEEG) field potentials were recorded using a 32-channel Neuralynx ATLAS system. Spike data from the same electrode recording with macroelectrode were acquired by bandpass filtering the raw signal from 600 to 9000 Hz. For the spike, the sampling rate was 32 kHz; the signals were referenced against one low-impedance reference electrode, and spikes were detected from the raw trace by applying a 30-μV threshold. Spike sorting was performed using *wave clus* (Quiroga et al., 2004). Once the sorting was completed, all clusters were categorized according to noise, multi-unit, or single-unit activity based on the waveform size, the shape of waveform compared with noise, the absence of power line interference, and an indication of a refractory interval through the procedures used in a previous study (Valdez et al., 2013). To visualize the spikes aligned with the memory task, a raster plot was created by aligning spike timestamps regarding to the timestamp for the visual stimuli presentation (bin size = 200 ms, time window = 1 s before and 3 s after stimuli). We then subdivided the entire prestimulus and during-stimulus periods into time-resolved windows using 200-ms sliding windows with 150-ms overlap (Urgolites et al., 2020).

### Unit identification by the prestimulus subsequent memory effect

We categorized pre-SME neurons that were statistically defined by comparing the distribution of each neuron’s firing rate (FR) similar to previously applied methods (Yoo et al., 2021). Neurons were classified as (1) “pre-SME” cells if the FR was higher in subsequently remembered words compared with subsequently forgotten words, (2) “anti-pre-SME” if the FR was higher to subsequently forgotten words relative to subsequently remembered words, or (3) “non-pre-SME,” if FR was not a significant response to recognition success. For the individual statistic test, we generated a *p*-value by identifying the position of the true rank sum statistic from the test applied to real data with 1000 rank sum values. Thus, permutation-based *p*-values were calculated to assess the significant difference between the conditions (i.e., remembered vs forgotten trials) in the prestimulus period by a null distribution of data samples. First, we collected the trials of the two conditions in a single set, then randomly partitioned the trials into two subsets. Next, we calculated the test statistics on this random partition and repeated steps (i.e., 1000 iterations) second and third analysis a large number of times and constructed a histogram of the test statistics (Maris and Oostenveld, 2007). Finally, we used the Wilcoxon signed-rank test for the group-level significant test and assessed the statistical significance of changes. All spike data analysis was conducted using the Statistics Toolbox in MATLAB software (version 2018b, MathWorks).

### Human participants

This study was approved by the Institutional Review Board of Seoul National University Hospital (H-1407-115-596). All participants provided written informed consent to participate in the study.

## Results

Memory and reaction time: results showed that patients remembered well as the mean frequency of correct responses across all trials was 80.0 ± 3.95%, mean ± SEM) and the average sensitivity, *d*, was 1.7 ± 0.27 (*p* < 0.05), which indicated that they had adequate memory. The overall reaction time for recognition was 1220.23 ± 239.73 ms for correctly remembered words and 1717.18 ± 885.66 ms for forgotten words (mean ± SEM).

### Firing rate of hippocampal and amygdala neurons

**Figure 3.**
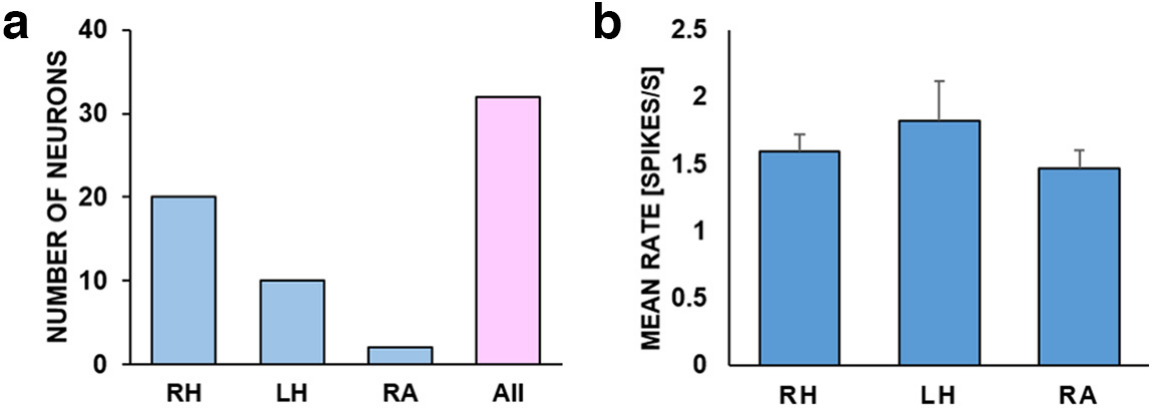
Numbers of neurons and mean firing rates from different brain areas. ***a***, Units’ numbers from each brain area were as follows, from left to right: 20, 10, and 2. ***b***, The mean firing rates of units, respectively. All units are included, regardless of firing rates. The mean firing rates of the hippocampal and amygdala are 1.71 and 1.47, respectively. Error bars are ±SEM. RH, right hippocampus; LH, left hippocampus; RA, the right amygdala.

We recorded spiking activities of single units in the hippocampus and amygdala as individuals performed word memory tasks. Thirty-two single neurons were isolated from the hippocampus and amygdala (30 from the hippocampus and two from the amygdala: 29 single units and three multiunits). Only single units that met the isolation criteria, including the interspike intervals (ISIs) ≤ 3 ms and the cluster number of neurons ≤0.5% (*n* = 29), were used in the final analyses. Ninety-three percent of most units (*n* = 27 out of 29) were regular-spiking neurons, and others were unclassified neurons (2/29) based on autocorrelograms (Barthó et al., 2004). Unit recordings were made from the hippocampal CA3 for patients 1 and 3. On average, the isolated single neurons fired 1.63 spikes per second. The mean firing rate of the hippocampal neurons was 1.71, which was higher than that of 1.47 in the amygdala (Fig. 3).

### Firing rate of encoding and activity

**Figure 4.**
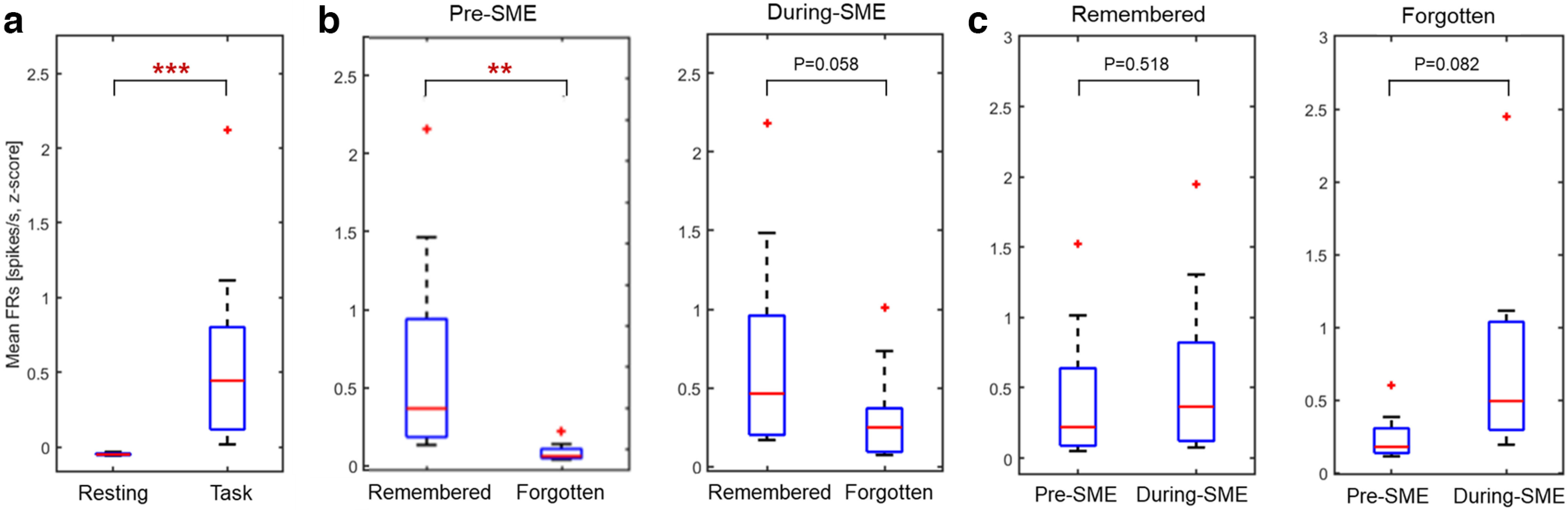
Group-level comparison of spiking activity in the hippocampus during an item memory task. ***a***, The mean firing rates across neurons (*n* = 11) during resting states versus memory encoding showed a significant increase in firing rates for memory encoding compared with resting states (Wilcoxon signed-rank test, ***p < 0.001). ***b***, Group-level comparison of pre-SME and during-SME hippocampal mean firing rates. During the preceding stimulus, hippocampal firing rates were higher for subsequent remembered than forgotten words (left, Wilcoxon signed-rank test, **p < 0.01). However, the trend during-SME hippocampal activity was not significant (right, Wilcoxon signed-rank test, *p* = 0.058). ***c***, Pre-SME and during-SME mean firing rates for an operation of words subsequently remembered (left) versus forgotten (right). The difference between pre-SME and during-SME was not significant in both remembered and forgotten words (Wilcoxon signed-rank test, *p* = 0.0518, *p* = 0.082, respectively).

We examined how the encoding activity during the presentation of words (i.e., task period) differed from the resting states. We analyzed the mean firing rates in the hippocampus during resting and memory encoding states for a given session. Results showed that the spiking activity for memory encoding was significantly higher than that for resting states. The mean firing rates across neurons during resting states versus memory encoding showed a significant increase in firing rates for memory encoding, which indicates that neurons exhibited task specificity (Fig. 4*A*, group-level analysis, Wilcoxon signed-rank test, *p* < 0.001).

### Firing rate of pre-SME predicts encoding success

**Figure 5.**
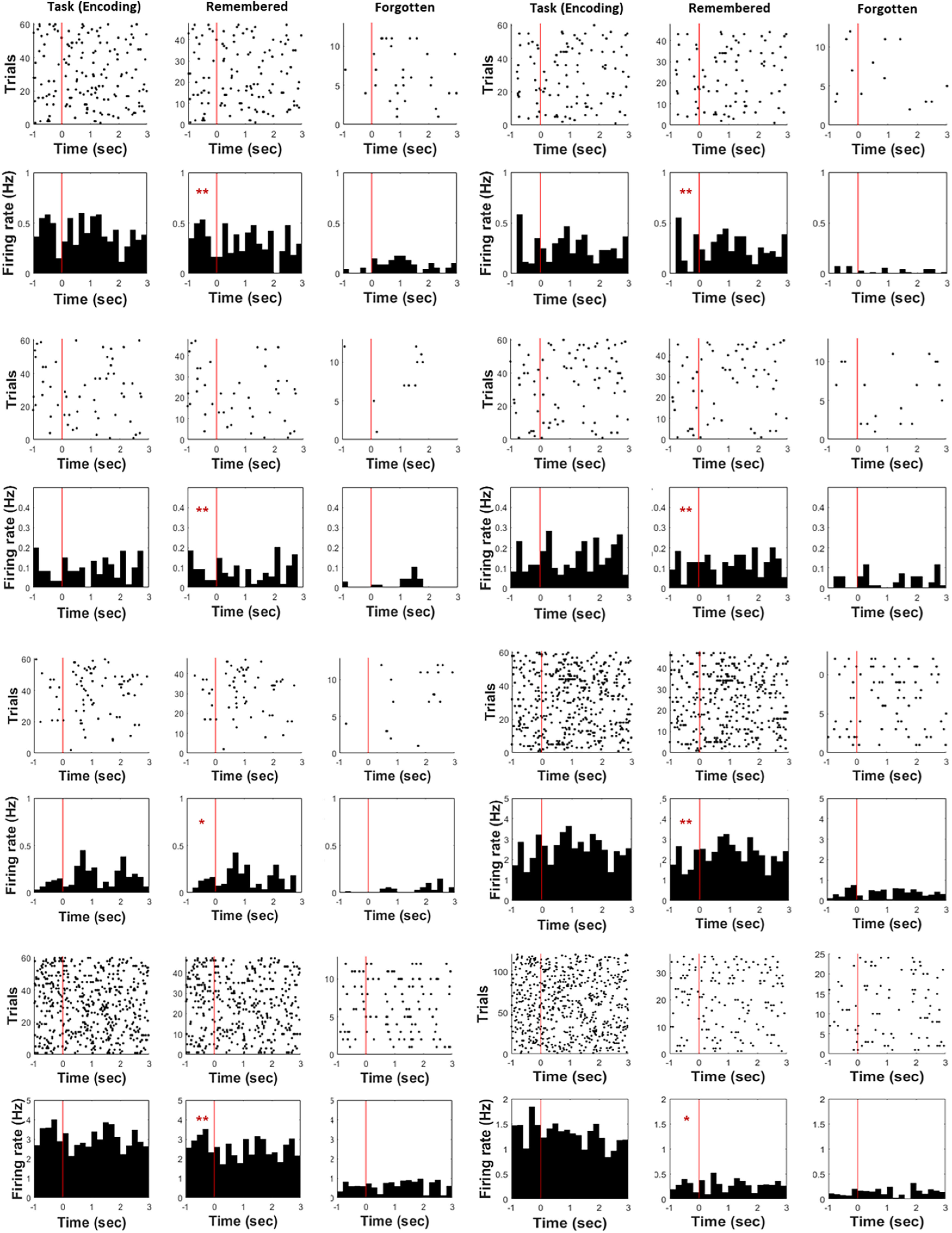
Hippocampal neuronal responses to subsequently remembered and forgotten word items. Shown are representative examples of hippocampal neurons that significantly increase firing rates across trials. Preceding stimulus and during-stimulus raster plot of firing rates during encoding sessions. The red lines mark stimulus onset at 0; the offset is at 3 s (*x*-axis). Raster rows represent single trials, and each dot represents an action potential. Next, the preceding stimulus and during-stimulus raster plot of firing rates for the remembered and forgotten conditions during encoding (**p < 0.01, *p < 0.05, permutation-based *p*-value).

To quantify the pre-SME, the mean firing rates were measured across the 1-s interval of the fixation cue period between remembered and forgotten trials. As we functionally defined proportions of each type of neuron, we then considered neurons as preceding stimulus activity if the firing rate responded to either (1) a fixation cross cue during the prestimulus interval and showed, or (2) a consistent elevated pattern of firing in all trials of that fixation cross. Behavioral classification of neurons indicated that 11 neurons (40%, 10 in the hippocampus, and one in the amygdala) showed a significant preceding stimulus activity and consistently increased firing rate in all trials in at least a 200-ms segment. These neurons exhibited significantly elevated FR during pre-encoding of successfully remembered compared with unsuccessfully remembered items, with consistently increased firing rate. The firing rates substantially differed before word stimulus onset corresponding to subsequent memory performance (pre-SME, *p* < 0.01, permutation-based *p*-value, shuffling 1000 times across units; Fig. 5, raster plot). Therefore, mean firing rates of neurons elicited by fixation cues preceding encoding words showed significantly increased patterns with the firing rates of subsequently remembered words compared with that of forgotten words. Three neurons showed a significantly decreased FR that predicted encoding success (anti-pre-SME). The rest 13 neurons did not show significant FR changes related to encoding success (non-pre-SME).

**Figure 6.**
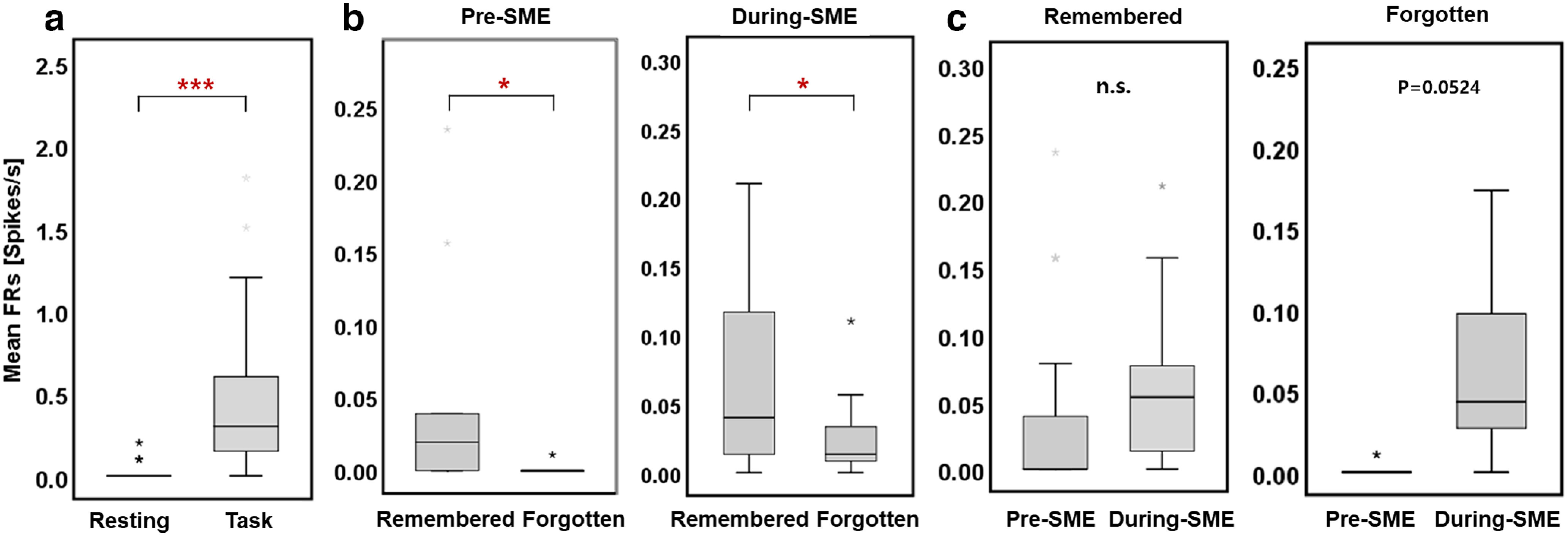
Hippocampal neuronal responses to subsequently remembered and forgotten word items. ***a***, The mean firing rates between resting states and encoding. Mean firing rates for the encoding duration are significantly higher than the resting state. ***b***, The hippocampal preceding stimulus neuronal activity (pre-SME) is significantly higher in subsequently remembered items compared with subsequently forgotten items (***p < 0.001, *p < 0.05, permutation-based *p*-value). This neuron was selective to only preceding stimulus with similar increases in activity in the during-stimulus condition. ***c***, The hippocampal spiking activity indicated the difference between preceding stimulus and during-stimulus activity was negligible in both remembered and forgotten conditions (n.s., *p* = 0.0524, respectively, permutation-based *p*-value). n.s. indicates not significant.

We compared the SME procedure between preceding stimulus and during-stimulus intervals. We found the hippocampal activity for subsequently remembered words was relatively larger than that for subsequently forgotten words before word stimuli (Fig. 4*B*, left, group-level analysis, Wilcoxon signed-rank test, df = 10, *p* < 0.01). Although the group-level analysis was not significant, hippocampal activity for subsequently remembered words was relatively larger than that for subsequently forgotten words after word stimuli (Fig. 4*B*, right, group-level analysis, Wilcoxon signed-rank test, df = 10, *p* = 0.058, see also Fig. 6 for a representative electrode site). These comparisons showed higher hippocampal neuronal activity for subsequently remembered versus forgotten words for both preceding and during-stimulus presentation. However, the spiking data showed no difference between the preceding stimulus and during-stimulus spiking activity in subsequently remembered and forgotten words (Fig. 4*C*, group-level analysis, Wilcoxon signed-rank test, *p* = 0.518, see also Fig. 6 for a representative electrode site). The difference was not significant, as the hippocampal spiking activity indicated merely a trend of higher during-stimulus spiking levels for the subsequently remembered words versus that of subsequently forgotten words. These results suggest that the during-stimulus activity was a continuation of the preceding stimulus activity, indicating that the during-stimulus neuronal activity was a prolongation of the preceding stimulus activity.

### First presentation effect of unit activity

To further examine how the preceding stimulus activity of the first presentation of word stimulus compares with the performance at the other presentation, we compared the mean spike activity from the first preceding stimulus activity with the mean spike activity from the other stimulus preceding activity. We showed that the first stimulus and other stimulus’s hippocampal preceding activity were higher for subsequently remembered but not for forgotten words (permutation-based rank sum test, *p* < 0.01), yielding hippocampal spiking activity before the presentation of predicted to-be-learned words.

### Spiking activity of task dependency and retrieval

**Figure 7.**
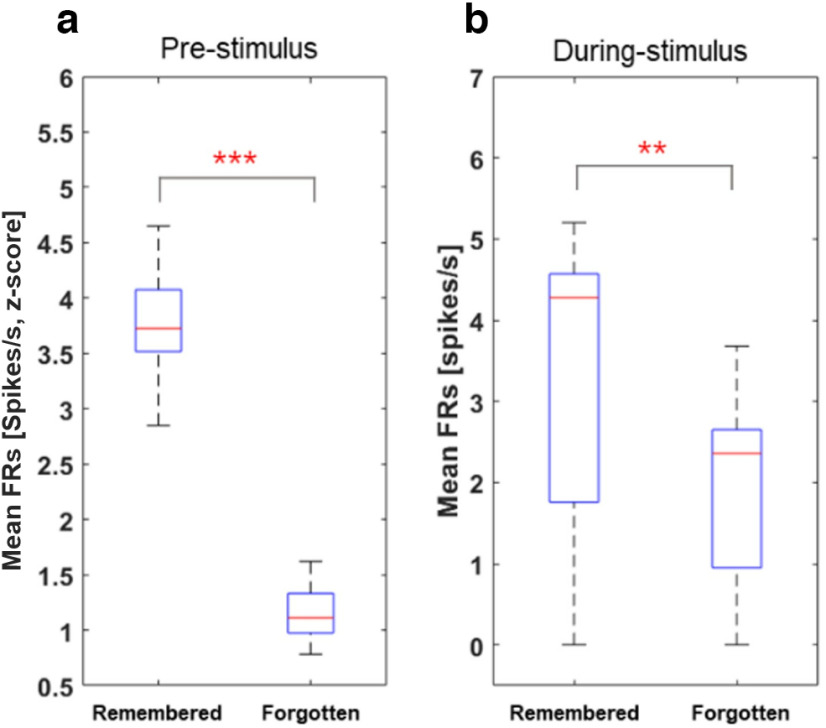
Group-level comparison of spiking activity in the hippocampus during an associative memory task. ***a***, Presubsequent memory effect (pre-SME) and (***b***) during-SME mean firing rates as an operation of words subsequently remembered versus forgotten words.

We also analyzed the group-level comparison of the associative memory task by an illustration in the item memory task, to confirm whether spiking activity would differ between the two different tasks (Jun et al., 2020). Results showed that the hippocampal activity in associative memory task also exhibited higher values for remembered words and lower in forgotten words either preceding stimulus or during-stimulus periods (Fig. 7, group-level analysis, Wilcoxon signed-rank test, *p* < 0.001 and *p* < 0.01, respectively). It indicates that the hippocampal preceding stimulus effect was exhibited in both conditions. To further study the relation between activity around the encoding and retrieval, we examined whether activity during the retrieval predicts memory performance on that stimulus. Results show that in retrieval, although during-stimulus hippocampal activity predicted whether memory performance would be correct (Wilcoxon signed-rank test, *p* < 0.001), the predictive effect was not observed in the preceding stimulus (Wilcoxon signed-rank test, *p* = 0.147).

## Discussion

In this study, we evaluated whether spiking activity preceding and during stimulus predicted subsequent successful memory formation in word memory tasks in patients with epilepsy. The hippocampal neurons, presented by a fixation cue preceding a word, increased the firing rates based on whether the word would be subsequently remembered. The during-stimulus activity also showed the trend of predicting subsequent successful memory formation. However, the firing rates were merely a prolongation of preceding stimulus firing rates in the item memory task. However, during retrieval, this predictive effect was not exhibited. These findings suggest that the hippocampal preceding stimulus activity during encoding may modulate successful memory formation and predict later memory success.

The brain activity that generates complex patterns even in the absence of sensory involvement or task is referred to as “prestimulus,” as opposed to activity following sensory input (Iemi et al., 2019). Studies exhibiting prestimulus activity have primarily investigated event-related potentials (ERPs), showing successful memory encoding (i.e., pre-SME). For example, neural activity in the prestimulus onset interval varied with items that were subsequently recalled or subsequently forgotten. It supports the idea that the preceding stimulus neural activity is sensitive to episodic memory performance (Klimesch et al., 1999; Fell et al., 2011; Gruber et al., 2013; Merkow et al., 2014; Salari and Rose, 2016). Although our data showed a similar hippocampal preceding stimulus effect in both word memory tasks, prior fMRI studies showed that pre-SMEs have been affected by task features, for instance, stimulus modality (Wagner et al., 1998a; Golby et al., 2001; Otten et al., 2006; Park and Rugg, 2010). Accordingly, it is considered to reflect, to some extent, the preparative arrangement of material-specific or task-specific and domain-general processes that influence memory performance.

What might be a rationale for modulating preceding stimulus hippocampal activity? One possibility is that neural activity reflects an attentional or preparative status in which the preceding stimulus cue recruits in anticipation of the upcoming stimulus. For example, the experimental task required participants to switch unpredictably from set up for a coming visual stimulus. In this case, an arrangement is needed to switch from the attentional concentration of the task fixation cue to the forthcoming stimulus. Therefore, hippocampal activity preceding stimulus was enhanced on those stimuli where processes arranged for the presentation of the fixation cue and resulted in a reasonably optimal preparative status. A possible neurochemical mechanism underlying these attentional shifts is the activation of the scheme by modulating the “reward value” of the preceding stimulus cue (Adcock et al., 2006), which described that the hippocampal pre-SMEs function together being activated in the ventral tegmental area, the origin of the mesolimbic dopaminergic system. Pre-SME in this system incites or elicits cues indicating the novelty of upcoming to-be-studied items (Wittmann et al., 2007). In this sense, hippocampal neurons may reflect the fixation cue operated by triggering the mesolimbic dopaminergic system, which may contribute to manipulating preceding stimulus hippocampal activity in conditions of novel study items.

Since the preceding stimulus and during-stimulus activity in encoding may be related, how would preceding stimulus activity regulate encoding eligibility? As depicted in Figure 4*C*, the raw spiking data revealed that during-stimulus firing, there is an extension of the preceding stimulus firing rates for each remembered and forgotten condition. In an earlier study, cumulative excitability in a single unit follows those units being partially influenced to correspond to a novel memory (Cai et al., 2016). More excitable units primarily activate with the upcoming items, and synaptic variations in those neurons create the memory trace (Rogerson et al., 2014). The neuronal firing of specific neurons in the hippocampal CA1 and other hippocampal subfields is critical in building a behavioral response that actuates “engram” neurons labeled when the cue is presented. This effect conjures up our experiences, and memories may be conveyed by simple ensemble activity without sequential structure (Dutta et al., 2020). Overall, the present study suggests that the more excitable neurons were already working at the time the item was presented and created well-built memory traces (Han et al., 2007).

Our findings reveal a cellular process in the human hippocampus representing preparatory activity and identify candidate neurons that may support the attentional shift for memory performance. Moreover, our results advance previous findings that the chance of an item being successfully encoded is related either to the pattern of neuronal activity stimulated by the item itself or to the neuronal activity that instantly heads (or that neuronal activity that immediately precedes) the item. Therefore, when the brain, specifically the hippocampal neurons, is gearing up to encode into episodic memory and hippocampal-mediated episodic memory, success may be augmented by high levels of preceding stimulus neuronal response.

The data indicated that words subsequently remembered induced more positive-going event-related potential (ERP) than words subsequently forgotten (Otten et al., 2006). It may reflect encoding attentional selection, providing an excitatory advantage to the hippocampal cells involved in coding the cue. Concerning the alternations in timing and expression of pre-SMEs, it is apparent that neuronal or oscillatory activity from iEEG field potentials is more sensitive than that from noninvasive measures for detecting pre-SMEs which may be missed with ERPs or BOLD signals. However, pre-SMEs using direct invasive electrophysiological studies need to be better understood. Our data provide neuronal evidence of pre-SMEs with a markedly higher temporal resolution, essential for noninvasive studies examining memory. These findings are applicable for the interpretation of studies employing indirect brain investigation [i.e., fMRI and electroencephalography (EEG)] of successful memory formation (Wagner et al., 1998b; Paller and Wagner, 2002; Otten et al., 2006) and an vital precursor to better understanding human memory. The present experiment investigated a small number of participants; hence, we cannot claim with any certainty that the statistical power was sufficient. Despite this limitation, we observed a consistent preparatory neuronal effect on each of the 11 neurons. In future research, we aim to recruit more extensive samples of patients and perform in-depth analyses considering variable hippocampal-subfield characteristics.

## Synthesis

Reviewing Editor: Christina Zelano, Northwestern University - Chicago Decisions are customarily a result of the Reviewing Editor and the peer reviewers coming together and discussing their recommendations until a consensus is reached. When revisions are invited, a fact-based synthesis statement explaining their decision and outlining what is needed to prepare a revision will be listed below. The following reviewer(s) agreed to reveal their identity: Salman Qasim.

The reviewers and I agree that this study has potential to contribute rare insight into human hippocampal function, and to our understanding of pre-stimulus brain states. The approach, including human single-unit recordings, is novel and the authors found evidence in support of their hypotheses. However, both reviewers raised substantial concerns about the statistics and felt that additional methods details were needed in order to assess the validity of the reported findings. See reviewers’ detailed comments which need to be addressed below:

Reviewer 1:

Here, the authors propose that increases in hippocampal firing rates preceding stimulus onset were predictive of stimulus memory in humans performing a word memory task, without reinstatement of this activity during retrieval. The motivation for this study is well-described, and the approach (human single-unit recordings) is novel and could provide a rare insight into human hippocampal function.

However, there are substantial weaknesses in the reported analytical and statistical methods that prevent me from assessing the validity of the stated conclusions. I thus cannot recommend the manuscript for publication in its current state. I describe these below and make prescriptive recommendations.

1. There is no statistical test or threshold provided to characterize significant increases in firing rate. This is critical, as the authors seem to cherry-pick 12 units showing “significant” activity preceding stimulus, but do not describe how this was determined relative to any null hypothesis of chance-level spiking. This is especially problematic as these 12 units are then analyzed at the group-level in Figures 4 and 5, making it difficult to assess these subsequent results.

a. To address this, the authors should provide a statistically determined threshold mean firing rate that these units exceed, or a statistical method for identifying timepoints at which the peak firing rate is significantly elevated.

b. A completely alternative (and, in my opinion, superior) approach might be to compute a true subsequent memory-effect as done in prior literature: compute a within-cell comparison of firing rate between remembered and forgotten trials, yielding a single test-statistic per cell. The authors do this just for Figure 3, but seemingly abandon the approach for categorizing “pre-SME” cells. An example of this approach is clearly demonstrated in PMID: 34742943.

2. More rigorous (permutation-based) statistics are required for between-condition comparisons to account for the possibility that the group results are caused by the very large difference in sample sizes between remembered and forgotten conditions that are exacerbated by the very small amount of data (1 second, low firing rates) analyzed.

3. In addition, due to the overall low sample size, it is critical that the authors provide raster plots of all of the neurons identified as having the identified pre-SME effects. The most compelling figure in this manuscript is Figure S2, where the authors provide one such example. This will build confidence in the results, which is important because the small sample size inherently limits certain statistical approaches that are norms in the field.

Reviewer 2:

This manuscript investigates preparatory hippocampal activity for memory encoding and tests whether neural firing prior to memory encoding predicts subsequent memory. The authors perform single neuron recordings in four patients with epilepsy during word memory tasks. The authors find evidence in support of their hypothesis. Overall, the manuscript may add to our understanding of pre-stimulus brain states but I suggest several revisions prior to publication.

1. This manuscript relies critically on the timing of activity, yet no information is provided as to how neural and behavioral data were synchronized. The authors should provide these details, along with any estimate of timing uncertainty (e.g. synchronization error)?

2. There are numerous grammatical mistakes throughout. I am sympathetic to those writing in a secondary language but I would nonetheless respectfully suggest they consult with a writing professional at their institution to clean up such errors.

3. The data come from a relatively small number of neurons (29 total). The authors then do “population” statistics on 12 units, which I am not sure is valid, particularly if unit isolation is imperfect and “neurons” are not truly independent. At the very least this should be mentioned as a study limitation.

4. Please provide example waveforms and or raw data to evaluate spike sorting and data signal quality.

5. Aside from Figure S2, I would prefer if the authors provided more single-trial raster plots for those neurons showing pre-stimulus and post-stimulus subsequent memory effects.

6. Hippocampal subfield segmentation with fMRI is notoriously tricky. The authors claim the recordings are from CA3 without providing sufficient detail to evaluate this claim.

7. Figure 5 suggests that pre-stimulus effects are larger than during-stimulus effects, which is very surprising.

Minor points

8. The authors should cite the substantial evidence of pre-stimulus (oscillatory) brain states on memory in the introduction, such as Guderian et al 2009. Other work using fMRI or during retrieval (work by Richard Addante, Juergen Fell and others) is also relevant and could be added.
